# Bradykinin’s carbamylation as a mechanistic link to impaired wound healing in patients with kidney dysfunction

**DOI:** 10.1186/s12915-025-02187-x

**Published:** 2025-03-12

**Authors:** Marta Kaminska, Urszula Kałucka, Janka Babickova, Małgorzata Benedyk-Machaczka, Eleni Skandalou, Melissa M. Grant, Hans-Peter Marti, Piotr Mydel

**Affiliations:** 1 Department of Microbiology, Jagiellonian University, Krakow, 30-387 Poland; 2 Broegelmann Research Laboratory, Department of Clinical Science, Faculty of Medicine, University of Bergen, Bergen, N-5021 Norway; 3 Department of Clinical Medicine, Faculty of Medicine, University of Bergen, Bergen, N-2021 Norway; 4 Institute of Clinical Sciences, University of Birmingham, Birmingham, B5 7EG United Kingdom; 5 Department of Medicine, Haukeland University Hospital, Bergen, N-2020 Norway

**Keywords:** Bradykinin, Wound healing, Carbamylation, Posttranslational modification

## Abstract

**Background:**

Uremic impairment of wound healing is a well-established phenomenon, however the etiology of this condition continues to be a medical enigma. Carbamylation, posttranslational modification (PTM) occurring with high frequency in uremic milieu, is known to have impact on structural and functional properties of proteins and peptides. Herein we show that carbamylation of the members of kinin-kallikrein system, that play an essential role in wound healing process, results in its aberrant functionality and impedes the complex process of tissue regeneration in uremic patients.

**Results:**

Through enzymatic assays we demonstrate that carbamylation of kininogen results in aberrant bradykinin generation. We confirmed that bradykinin is efficiently carbamylated in uremic conditions and, alternatively, by activated neutrophiles. Moreover, this modification affects proteolytic cleavage of the peptide, potentially leading to the accumulation of the carbamylated form. Modified peptide demonstrated lower affinity toward its receptors. Carbamylation diminished bradykinin’s ability to stimulate expression of the B_1_ receptor and cytokines essential in wound healing process. Carbamylated bradykinin was significantly less potent in promoting angiogenesis and keratinocyte motility as compared to the native form. In the in vivo murine model of wound healing, we observed impaired collagen fiber production and delayed re-epithelialisation in the presence of carbamylated form.

**Conclusions:**

Carbamylation-driven impairment of wound healing is a mechanistic link to wound persistence in uremia. Importantly, production of carbamylated bradykinin in localized inflammatory milieus could be a significant contributor to delayed wound healing and formation of chronic wounds in diabetes or psoriasis.

**Supplementary Information:**

The online version contains supplementary material available at 10.1186/s12915-025-02187-x.

## Background

The negative impact of uremia on wound healing has been well acknowledged in animal research and clinical medicine for more than half a century. First reports published as early as 1960s have described the adverse effects of uremia on fibroblast proliferation and collagen production in wounds [1]. In animal models, poor wound healing during chronic kidney disease has been shown to be a multifactorial pathology, but it is at least partially due to the accumulation of uremic toxins. These metabolites may induce the underlying chronic inflammatory state, and have been shown to be responsible for hemostasis defects, disruption of keratinization kinetics, delayed rate of granulation [2] and vascularization, with overall slower rate of cell proliferation [3].

Uremia, the clinical condition associated with end-stage renal disease, results from the retention of waste products that, under physiological conditions, should be cleared by the kidneys. Once these uremic toxins accumulate, they exert devastating effects on all organ systems [4]. Urea is quantitatively the most abundant retained solute in the body, where it is in equilibrium with the reactive metabolite cyanate [5]. Cyanate reacts with amino groups of N-termini or side chains of lysine residues of proteins and peptides, resulting in the formation of carbamyl residues [6]. Cyanate concentrations are estimated to be over 25 times higher in end-stage renal disease patients than in healthy individuals [7, 8], resulting in a more frequent generation of carbamylated molecules. Elevated serum cyanate concentrations have a major impact on proteins, leading to the loss of local amino acid residue charge, increased hydrophobicity, and finally structural and functional changes, including impaired interactions with other proteins, as has been previously observed in case of matrix metalloproteinase-2 (MMP2) [9], LL-37 [10] and insulin [11]. As this modification occurs non-enzymatically, it can affect all targets exposed to cyanate ions, including peptides and proteins regulating tissue repair.

Wound healing is a dynamic process involving complex interactions between extracellular matrix molecules, soluble mediators, resident cells and infiltrating leukocytes, with the immediate goal of restoring the integrity and homeostasis of the tissue [12]. Wound healing is regulated by a number of factors, among them the kinin-kallikrein system (KKS) [13]. Kinins, including bradykinin (BK), are vasoactive peptides that act as key modulators of pain [14], vasodilation, vascular permeability [15], inflammation and wound healing. Upon release from high molecular weight kininogen (HMWK) by plasma kallikrein (pKLK), BK’s biological effects are exerted via the interplay between the constitutive kinin B_2_ receptor (B_2_R) and the inducible kinin B_1_ receptor (B_1_R). BK can stimulate keratinocytes to express antimicrobial peptides, various pro- and anti-inflammatory cytokines and to proliferate. At the same time extracellular matrix remodeling is initiated by the MMPs production [16] and tissue vascularization is induced by VEGF release in response to BK stimulation. The combined effort of dermis and epidermis cells is essential for proper granulation and the restoration of the epithelial barrier. If any stage of this process is disrupted by, for instance, carbamylation of KKS components, wound healing will be delayed, and that in turn could lead to the formation of chronic wounds.

We hypothesized that high level of urea-derived cyanate leads to carbamylation-triggered structural alterations within the epithelial milieu, disrupted functioning of regulatory systems such as KKS, finally resulting in the impaired wound healing in chronic kidney disease patients. Therefore, in this study we examined how carbamylation will affect the generation of this peptide from HMWK, bradykinin’s downstream metabolism by ACE1, NEP and CPM proteases and its biological activity: its affinity to B_1_ and B_2_ receptors, production of recognized wound healing mediators (cytokines and proteases), promotion of keratinocyte re-epithelialization and angiogenesis in in vitro models and keratinization in an in vivo wound healing murine model.

## Results

### Carbamylation affects BK processing

**Fig. 1:**
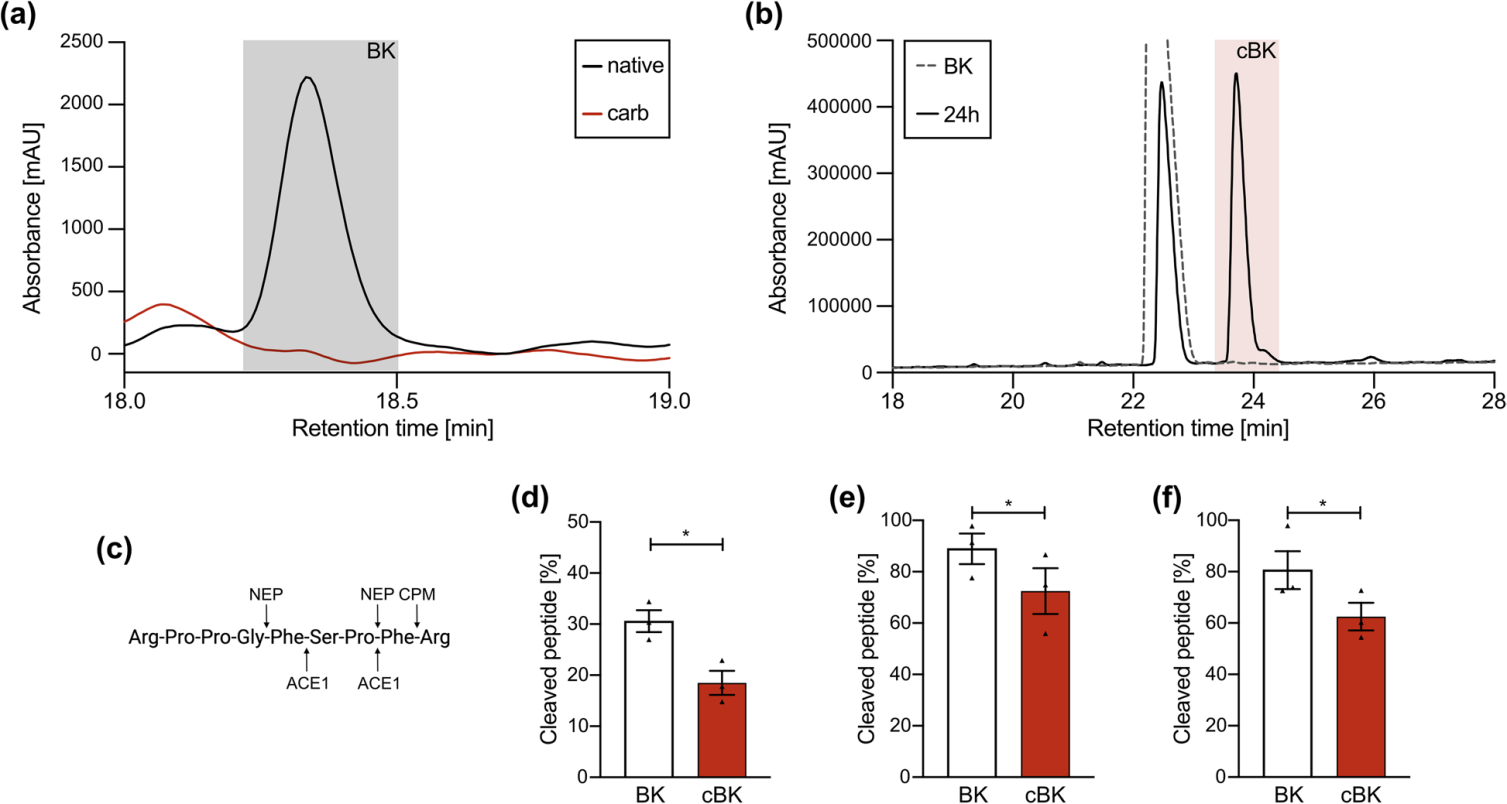
cBK’s enzymatic generation and turnover after carbamylation. **A** BK (black box) release from native (black line) or carbamylated HMWK (red line). **B** Generation of cBK (red box) during peptide incubation with PMA-activated granulocytes, with dotted line indicating BK load control. Representative result. **C** BK cleavage sites of CPM, NEP and ACE1. BK and cBK were cleaved by (**D**) CPM, **E** NEP or (**F**) ACE1, and reaction products were separated via HPLC (*n* = 3, biological replicates). Results are shown as % of cleaved peptide ± SEM when compared to respective loading control – BK or cBK. Statistical significance was evaluated with paired t-test, * if *p* < 0.05, ** if *p* < 0.01; *** if *p* < 0.001

HMWK’s carbamylation abolishes the pKLK-driven generation of BK (Fig. 1A). N-terminal of BK may be additionally efficiently carbamylated by two mechanisms: exposure to cyanate ions and exposure to PMA-activated granulocytes in myeloperoxidase-dependent manner (Fig. 1B). Subsequently BK is degraded by a group of proteases: NEP, ACE1 or CPM (Fig. 1C). Although all three preferentially cleave peptides or dipeptides at the C-terminal, we observed significant interference with substrate recognition upon N-terminal modification of BK for all three of the examined proteases: cBK turnover was slowed down by 12% for CPM, NEP – 16% and ACE1 – 18% (Fig. 1D, E, F, respectively) when compared to BK. This data shows that MPO-driven BK modification will significantly dysregulate its enzymatic turnover, leading to the accumulation of carbamylated bradykinin.

### cBK is a weaker agonist for both B_2_ and B_1_ receptors

**Fig. 2:**
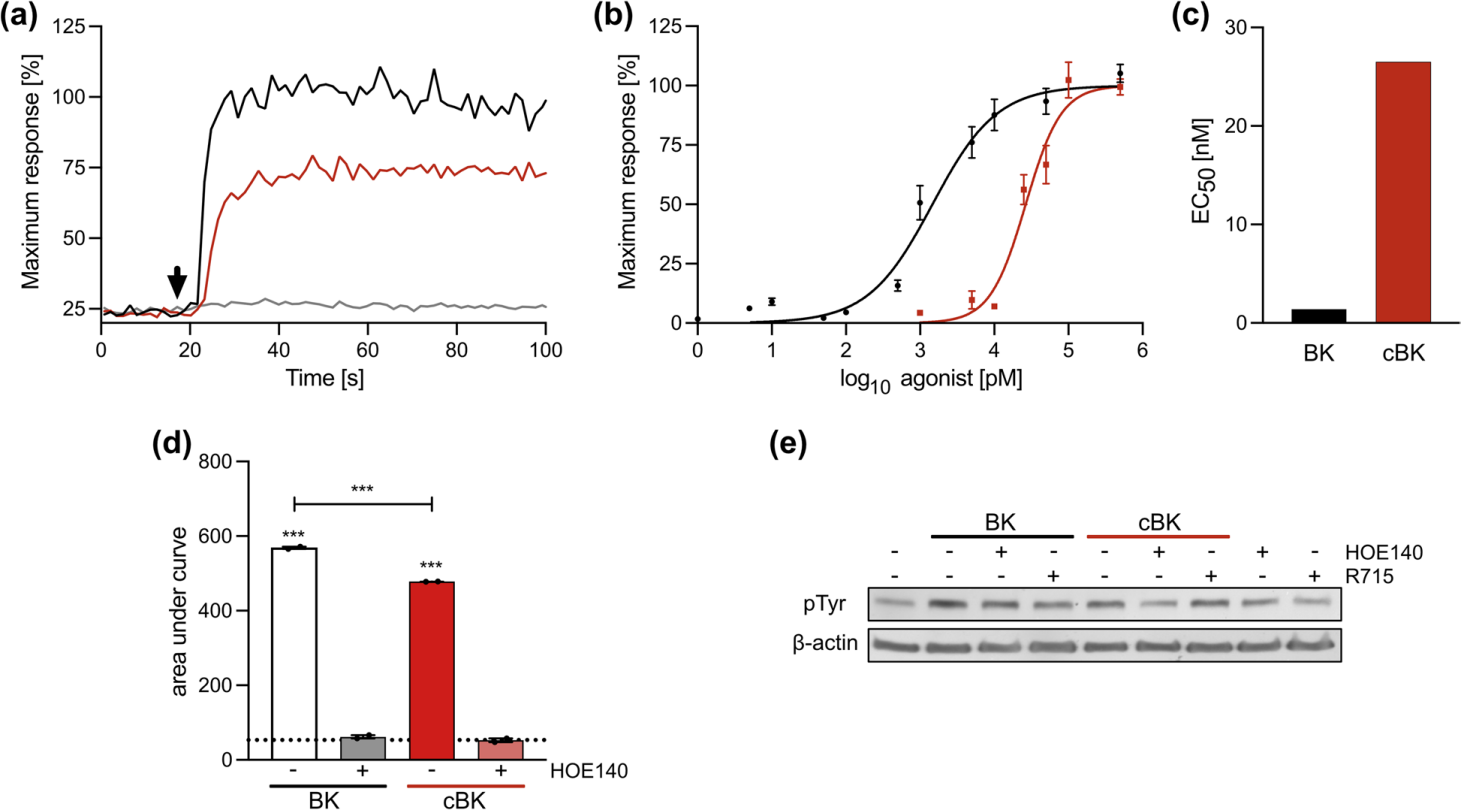
Carbamylation affects BK’s receptor affinity: cBK is a weaker agonist for B_2_ and B_1_ receptor. HaCaT cells were stimulated with native (BK, black) and carbamylated (cBK, red) peptide. **A** HaCaTs were stimulated with 100 nM of peptides at 17 s (black arrow) after measurement start. Representative result. Results were calculated as relative fluorescence units (RFU) fold change from the signal recorded prior to stimulation and recalculated to % of maximum observed response (to fetal bovine serum (FBS)). **B** Non-linear regression analyses were performed to generate dose–response curves and calculate EC_50_ using agonist vs. response equation (*n* = 6, biological replicates). **C** EC_50_ values for BK and cBK. **D** Cell response to 100 nM BK/cBK stimulation in presence/absence of 3 µM HOE140 (*n* = 2, biological replicates). **E** pTyr residues were detected in HaCaT lysates post 500 nM BK or cBK stimulation for 15 min in the presence/absence of B_2_ (HOE140) or B_1_ (R715) receptor inhibitors. Blots were then probed for β-actin as a load control. Representative result. Results are expressed as mean ± SEM. Statistical significance was calculated with One-way ANOVA with Dunnett and Sidak post hoc. *** if *p* < 0.001

Due to slower turnover contributing to accumulation of the cBK in the tissue, we next took a closer look at its biological effects. Firstly, we investigated impact of carbamylation on BK’s affinity to its receptors: B_1_ and B_2_. As the B_2_ receptor signals through calcium mobilization, its activation was evaluated using HaCaT cells loaded with calcium sensitive dye. The B_2_-stimulatory potential of 100 nM cBK was lower by over 25% than in case of the response elicited by the native BK (Fig. 2A). The half maximal effective concentration (EC_50_) of BK was increased 17-fold upon its carbamylation (26.55 ± 0.0011 nM for cBK, 1.47 ± 0.0012 nM for BK) (Fig. 2B, C). The observed effect is B_2_ receptor-specific, as the presence of B_2_ inhibitor (HOE140) blocked the intracellular calcium influx (Fig. 2D).

Next, we evaluated the activation of both B_1_ and B_2_ receptors via detection of pTyr residues in HaCaT lysates. The intensity of the observed band (estimated molecular weight 140 kDa, corresponding to the mediator PLCy) [17] was significantly lower after stimulation with cBK (Fig. 2E, for blot images see Additional file: Fig. S1-2), and the signal was further reduced by introduction of B_2_-specific inhibitor. In contrast, B_1_-specific inhibitor (R715) only had moderate effect after BK stimulation. No decrease in pTyr levels were observed in samples treated with both cBK and R715, the B_1_ receptor inhibitor. These results clearly indicate that carbamylated bradykinin is a markedly weaker agonist for B_2_ and this loss of activating potential is even more pronounced for the B_1_ receptor, likely affecting the downstream effects [18].

### Carbamylation of BK impedes expression of wound healing mediators

**Fig. 3:**
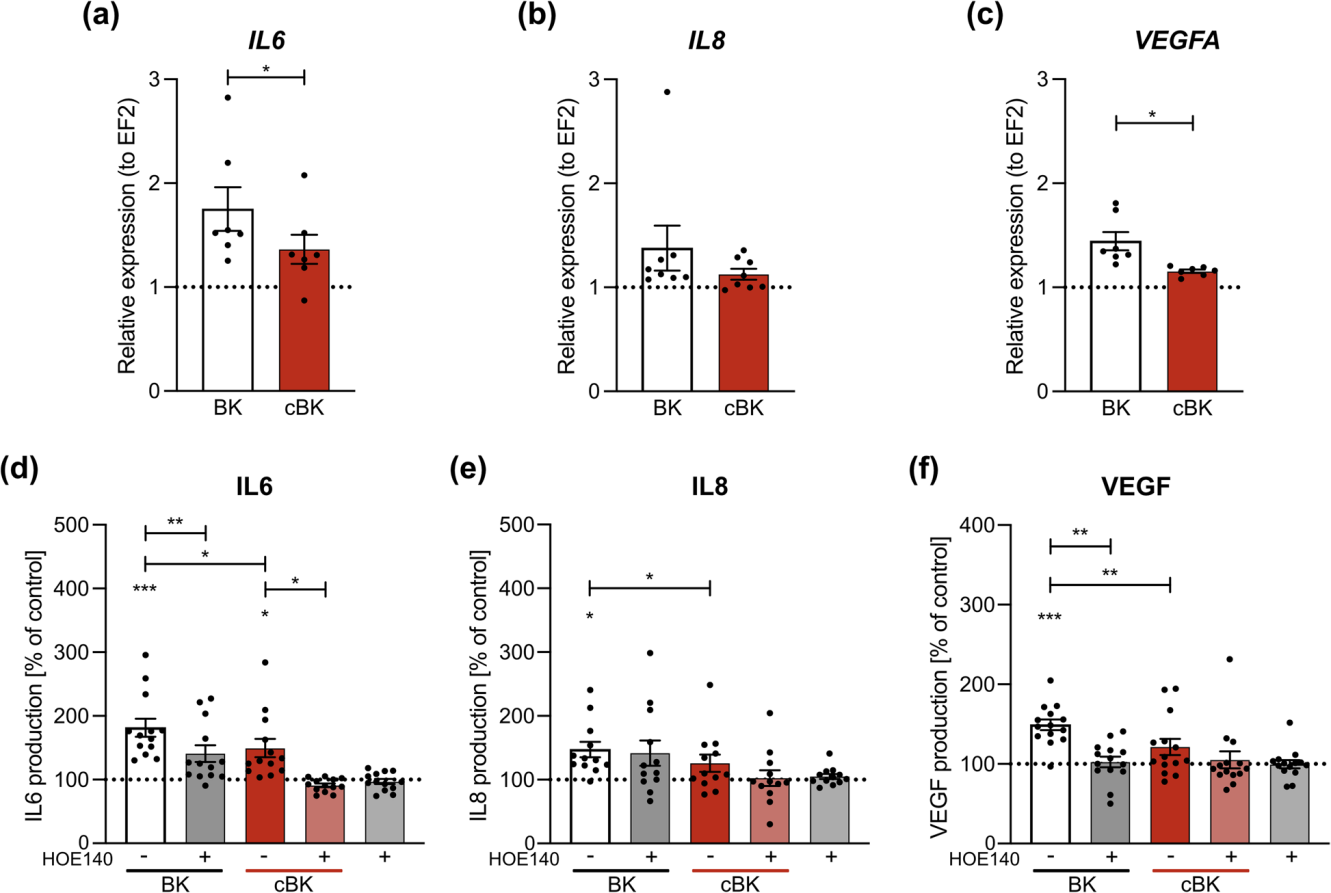
HaCaT cytokine production upon stimulation with BK (black, dark grey) or cBK (red, light red). After 4 h mRNA levels of (**A**) IL6 (*n* = 7, biological replicates), **B** IL8 (*n* = 8, biological replicates) and (**C**) VEGF-A (*n* = 7, biological replicates) were assessed with qRT-PCR. Results are presented as mean ± SEM. Statistical significance was calculated with the paired t-test. After 24h, levels of secreted (**D**) IL6 (*n* = 7, biological replicates), **E** IL8 (*n* = 7, biological replicates) and (**F**) VEGF (*n* = 7, biological replicates) post-BK stimulation in presence/absence of HOE140 were measured using ELISA. Results are presented as mean ± SEM. Statistical significance was calculated with the one-way ANOVA with Sidak post hoc. * if *p* < 0.05; ** if *p* < 0.01; *** if *p* < 0.001

Wound healing process is regulated, among others, by mediators of proliferation or angiogenesis. As BK has been previously reported to induce the expression of several acute wound healing cytokines (IL6, IL8) [19] and growth factors (VEGF) [20], we investigated whether carbamylation affects BK’s ability to trigger the cytokine expression at mRNA (Fig. 3A-C) and protein level (Fig. 3D-F).

When compared with the effect exerted by native BK, cBK failed to efficiently induce expression of *IL6* and *VEGF* at mRNA levels (Fig. 3A and C, respectively). Similar change was observed at the level of secreted protein, where upon cBK stimulation IL6 expression level was lower by 41% than during stimulation with BK (Fig. 3D), while VEGF was produced at a rate lower by 28% (Fig. 3F). Induction of *IL8* was not significantly different at mRNA level (Fig. 3B), the difference became significant at the protein level, with secretion induced at a rate lower by 17% upon stimulation with cBK than BK. Interestingly, IL6-inducing activity of cBK was completely abolished by the B_2_ receptor block (Fig. 3D). These results demonstrate that among the downstream effects impaired by cBK’s loss of affinity towards the B_1_ and B_2_ receptors is the lower production of cytokines, which in turn could hamper processes such as cell migration, proliferation or angiogenesis.

### Impact of cBK on B_1_R and B_2_R expression

**Fig. 4:**
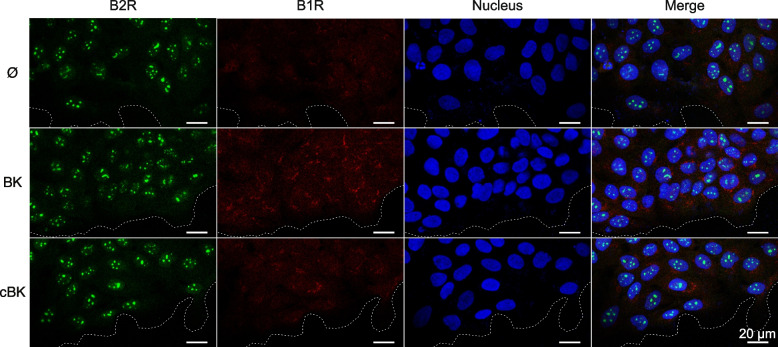
Expression of B_2_/B_1_ receptors at the gap edge in the in vitro wound healing model. After removal of the insert HaCaT cells were incubated without (top panel), with 500 nM BK (middle panel) or cBK (bottom panel) for 24 h. Cells were then immunostained against B_2_ (green) and B_1_ (red) receptor. DAPI (blue) was used as a nuclear counterstain. Wound edge has been additionally marked with a dotted line (white). Representative images. Scalebar 20 µm

Since we noted significant alterations in receptor activation upon stimulation, we wanted to examine if the receptor expression and cellular localization would be affected by stimulation with the cBK. In the HaCaT wound healing model, B_2_R’s levels remained unchanged (Fig. 4), whereas B_1_R levels were significantly increased in cells at the wound edge stimulated with both native and carbamylated peptide. Notably, this effect was less prominent after stimulation with cBK and the receptor appears to cluster in the center of the cells, in the proximity of nucleus. The receptor B_2_ is expressed constitutively and is not degraded upon stimulation. Interestingly, the presence of the nuclear B_2_R was prominent. On the other hand, B_1_ receptor’s expression is stimulation-dependent, and lower for cBK, which could contribute to the observed cytokine production dysregulation and delay the potential wound healing response.

### cBK retains the potential to stimulate MMP9 expression

**Fig. 5:**
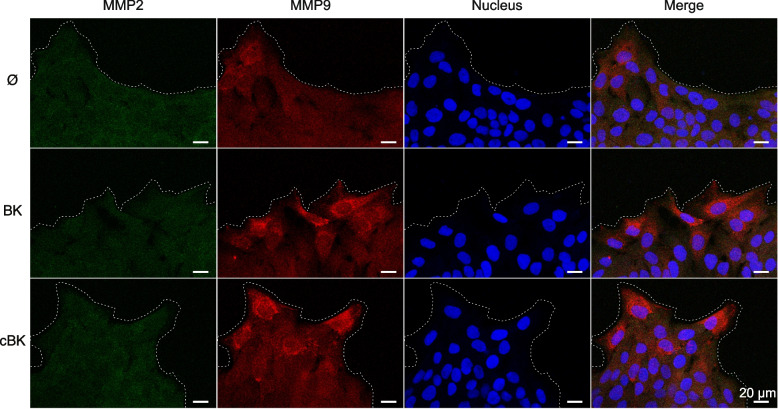
Expression of MMP2/MMP9 at the gap edge in the in vitro wound healing model. After removal of the insert HaCaT cells were incubated in absence of BK (top panel), with 500 nM BK (middle panel) or cBK (bottom panel) for 24 h. Cells were then immunostained against MMP2 (green) and MMP9 (red). DAPI (blue) was used as a nuclear counterstain. Wound edge has been additionally marked with a dotted line (white). Representative images. Scalebar 20 µm

MMP2 and MMP9, essential in remodeling phase of wound healing, have been shown to be upregulated in response to various kinins [21]. Using confocal microscopy, we have observed that the expression of MMP2 remained low regardless of the stimulant, while MMP9 increased significantly in the motile keratinocytes (protrusions at the wound edge) upon stimulation, although both peptides were equally efficient in triggering this response (Fig. 5).

### Effect of BK carbamylation on migration and angiogenesis

**Fig. 6:**
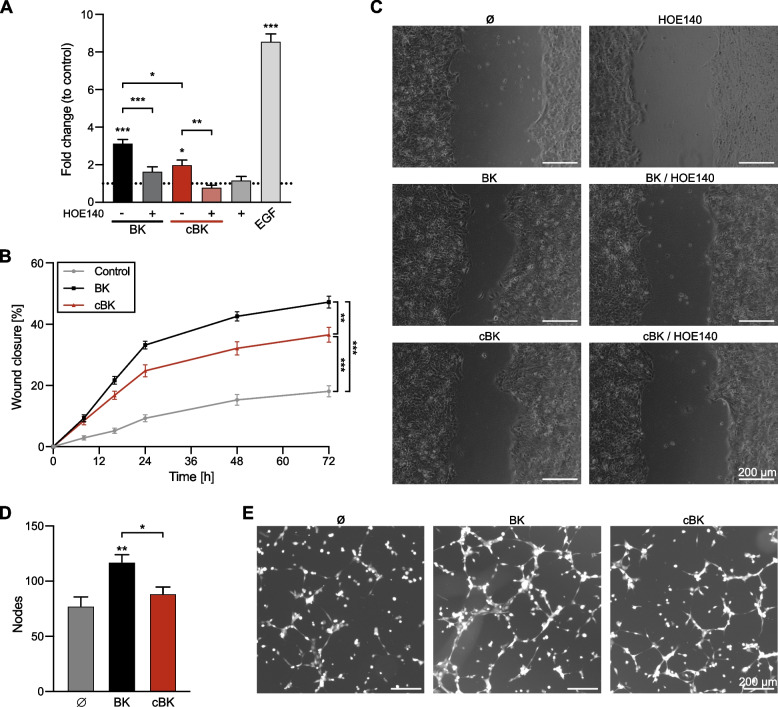
Gap closure and angiogenesis after stimulation with native (BK, black) and carbamylated (cBK, red) peptide. **A-C** After removal of the insert HaCaT cells were incubated with 500 nM BK, cBK or 12.5 ng/ml EGF, with or without 2 µM HOE140. Wound closure was monitored for up to 72 h. **A** Cell migration after 24 h of stimulation with peptides shown as mean relative fold change ± SEM. (*n* = 3, biological replicates) (**B**) Cell migration kinetics after stimulation with BK and cBK shown % of wound closure mean ± SEM. (*n* = 3, biological replicates) (**C**) Wound closure after 72 h of incubation. Scalebar 200 µm. Representative images. **D-E** CFSE-stained HBEC-5i cells were incubated in the presence of 0.5% FBS with or without 1 µM BK or cBK. Angiogenesis was recorded at 4h 30min, and results are presented as tube nodes mean ± SEM. (*n* = 4, biological replicates). Scalebar 200 µm. Statistical significance was calculated with the One-Way ANOVA with Dunnett and Sidak post hoc. * if *p* < 0.05; ** if *p* < 0.01; *** if *p* < 0.001

We next examined downstream elements of the remodeling phase: keratinocyte migration and endothelial angiogenesis. Monolayer of HaCaT cells was allowed to form, and then closure of gaps generated by insert removal was monitored for 72 h (Fig. 6A-C). At 24 h, a significant increase in cell migration in response to BK and cBK was observed (3- and a twofold respectively) (Fig. 6A). Over the course of 72 h, stimulation with BK-induced migration to a higher degree than was observed in the presence of cBK, but in both cases keratinocytes migrated faster than the control cells (Fig. 6B). Presence of B_2_R inhibitor strongly hampered BK’s activity, while it completely abolished migration in the presence of cBK (Fig. 6A and C). This indicates that cBK is able to promote keratinocyte migration only through the B_2_R, which significantly decreased it potential to stimulate wound closure.

Next, microvascular endothelium was cultured in GelTrex with BK or cBK. HBEC-5i cells stimulated with BK showed nearly 52% increase in node formation when compared to unstimulated controls. Carbamylation of the peptide abolished its pro-angiogenic activity (Fig. 6D-E), which could have adverse impact on the re-vascularization of the wounded area.

### cBK impedes collagen production in vivo

**Fig. 7:**
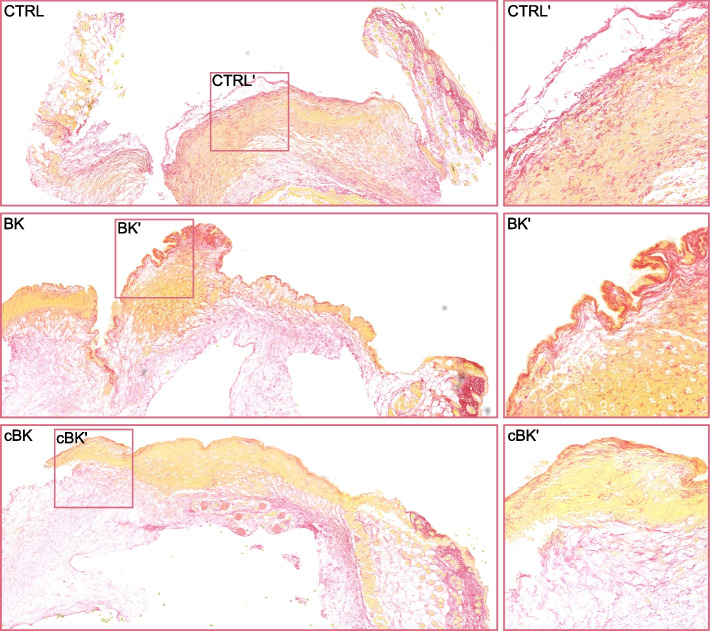
Wound healing and collagen fiber organization post-treatment with BK/cBK in the in vivo mouse model. Wounds were injected with 10 µl of 100 nM BK, cBK or PBS. Samples for collagen fiber imaging were collected after 24 h and tissues were stained with picro-Sirius red. Representative images

Migrating fibroblasts produce a stronger, organized matrix of collagens and proteoglycans, gradually replacing loose and highly hydrated temporary matrix. To evaluate the impact of cBK on collagen fibers’ production we employed murine wound healing model, in which saline, BK or cBK were injected cutaneously at the wound periphery and the tissue for histopathological examination was collected after 24 h. Tissue from sites treated with BK were characterized by distinctly higher organization of collagen fibers (red) (Fig. 7), particularly at the wound surface. Treatment with cBK significantly impeded collagen production as compared to untreated animals showing sparse fibers predominantly at wound surface, and limited amount dispersed through the tissue. Carbamylation of BK clearly alters the production and deposition of extracellular matrix in the wound milieu, which in turn could contribute to a slower re-epithelialisation.

## Discussion

Multiple evidence show how the kininergic system is able to modulate the wound healing process [21–23]. Although it is well established that tissue repair is substantially impaired over the course of chronic kidney disease and uremia [24–26], to date no report described the direct impact of carbamylation on KKS effector functions. Herein we propose that carbamylation of the N-terminal amino group of BK might represent a mechanistic link between uremia and dysfunctional wound healing in hemodialysis patients.

Bradykinin is released from the HMWK through cleavage by pKLK, a process we have shown to be impaired by kininogen carbamylation. The most probable reason for this observation is potential carbamylation of lysin K380 at the P1 of the cleavage site, with BK’s N-terminal arginin at P1’ position [27]. BK is efficiently modified by neutrophil myeloperoxidase-derived cyanate and regardless of incubation time or cyanate concentration, only a single amino group (the N-terminal) was found to be carbamylated. This modification increases peptide’s hydrophobicity as was previously observed in case of insulin [11] or LL-37 [10]. Released bradykinin has a relatively short half-life in plasma, however, to the best of our knowledge it’s half-life in the skin is yet to be investigated. When it comes to the cardiovascular tissue, where BK’s vasoactive functions are of special interest, its half-life in aorta is significantly extended [28], as is also observed in patients treated with ACE1 inhibitors [29]. Here, we demonstrate that carbamylation is another mechanism by which BK’s half-life may be extended. Upon modification of its N-terminal amino group, this peptide loses its affinity to its major proteolytic deactivators, including ACE1, NEP and CPM [30, 31]. This could contribute to the accumulation of cBK in the wound milieu, and drastically lower generation of BK’s derivatives, such as the high affinity B_1_R ligand, generated by CPM – des-Arg^9^-bradykinin [32]. Other BK metabolites (BK 1–7 and BK1-5, and BK1-4 – products of ACE1 [33] and NEP [34] activity, respectively) are not B_1_ and B_2_ receptor ligands. While BK1-4 is not an active metabolite, BK1-7 and BK1-5 reportedly retain their vasodilatory activity [35], and they have been implicated in the process of thrombin-induced platelet aggregation inhibition. Therefore, carbamylation may contribute to the disruption of hemostasis during early stages of wound healing.

The N-terminal of BK has been proposed previously as key in binding to both B_1_R and B_2_R [36]. Carbamylation, leading to N-terminal modification and increase in hydrophobicity of peptide, impeded activation of both receptors. We show that cBK activates B_2_R at a significantly lower level, resulting in a reduced calcium influx, a characteristic sign of this receptor’s activation in keratinocytes [37]. Both B_2_- and B_1_-induced signaling pathways converge downstream, which can be observed through, for example, phosphorylation rate of phospholipase C-γ [17]. Our results indicate that, while BK activates both B_1_R and B_2_R, cBK is only able to activate B_2_ – and this has been observed throughout this study’s evaluation of different effector functions of BK. This may be due to a cumulation of loss of affinity to cell surface-CPM and to B_1_R itself due to lack of N-terminal charge.

BK’s biological activity has been investigated in a number of cell lines and primary cells, such as airway smooth muscle cells [38, 39] or lung fibroblasts [19]. There BK induces expression of molecules vital in mediating wound healing processes: IL6, IL8 and VEGF. We observed similar effect using keratinocytes, once again noting weaker stimulatory potential of cBK than that of BK. The presence of B_2_R inhibitor did not fully abrogate the IL6 and IL8 upregulation, clearly indicating contribution of B_1_R in these cytokines’ production. BK-driven VEGF expression appears to be mediated solely through B_2_R, in line with previous findings. In IL6-deficient mice [40], as well as in Cxcr2-knockout mice [41], lack of these key mediators impaired neutrophil infiltration and neovascularization. This carbamylation-driven decrease in mediators’ production observed herein could impact efficient inflammatory response and subsequent wound closure.

Similarly, BK’s potential to induce production of B_1_R was hampered by carbamylation. Insufficient B_1_R involvement in the wound healing can be detrimental to the initial, inflammatory phase of the process, affecting especially neutrophil infiltration rate [42] and lead to delay in re-epithelialization [43]. Keratinocyte migration is essential in restoration of the epithelial barrier during wound healing, and it is facilitated by extracellular matrix remodeling catalyzed by, among others, matrix metalloproteases [12]. Albeit we observed no alterations in MMP production, BK carbamylation hampered downstream wound healing processes: collagen production, cell migration and angiogenesis. Migration of the HaCaT cells was significantly delayed in the presence of cBK as compared to native peptide and formation of the new nodes in the angiogenesis process was abolished completely. Previous reports have attributed full BK-driven stimulation of migration solely to the B_2_R signaling, and herein addition of B_2_R inhibitor also completely abolished effect exerted by the cBK. In case of angiogenesis, BK has been shown to directly stimulate endothelial cell proliferation and NOS upregulation [44], and to potentiate VEGF production by tissue resident fibroblasts [20, 45]. B_1_R is a crucial mediator of NOS activation, and bradykinin’s loss of affinity to B_1_ due to carbamylation could explain its significantly lower angiogenesis-stimulatory potential observed in this study. Finally, in murine model we have shown that cBK efficiently inhibits early production of collagen fibers and re-epithelialisation. Similar phenomenon was documented previously using B_1_/B_2_ receptor knockout murine models [43], where lack of B_2_ receptor impaired both collagen production and also re-epithelialization. Lack of either B_1_ or B_2_ hampered immune cell infiltration into the damaged area [43], which could present yet another mechanism for BK-driven delayed return to homeostasis in carbamylating microenvironment.

## Conclusions

In conclusion, we show that carbamylation of proteins and peptides may be a mechanistic link between uremia and dysfunctional wound healing in end-stage renal disease, as it affected crucial stages of BK’s functionality: from efficient peptide turnover, loss of B_2_ and B_1_ receptors affinity, and finally impaired keratinocyte migration, collagen production and angiogenesis. This could have detrimental impact on well-being of patients suffering not only from uremia, but also systemic or local states of chronic inflammation, such as diabetes or psoriasis, contributing to formation of persistent wounds.

## Methods

### Peptide cleavage

Ten µg of BK or cBK (ProImmune) were incubated with 15 ng of CPM (RnD; 1 h in 50 mM MES buffer pH 6.0 containing 5 mM CaCl_2_, 0.2% Triton-X100), 20 ng of NEP (RnD; 2 h in 50 mM HEPES buffer pH 7.4 containing 150 mM NaCl, 0,01% Triton-X100) or 40 ng of ACE1 (Merck; 4 h in 20 mM HEPES buffer containing 100 µM ZnCl_2_, 300 mM KCl) in final volume of 11 µl in 37 °C. Substrate controls were incubated in parallel without enzymes. Five µg of HMWK (Merck) were incubated with 500 nM of pKLK (Merck; 24 h in 50 mM Tris–HCl buffer pH 7.5 containing 100 mM NaCl, 0.01% Triton-X100) in final volume of 35 µl in 37 °C. Reactions were stopped with addition of TFA, TCA, EDTA and inhibitors for ACE1 (1 µM lisinopril), NEP (4 mM thiorphan), pKLK (1 mM AEBSF), and analysed via HPLC.

### MPO-driven BK carbamylation

Peripheral blood was obtained from healthy, de-identified volunteers and granulocyte/erythrocyte fraction was isolated using Lymphoprep™ (Serum Bernburg AG) following the manufacturer’s instructions, suspended in 1% polyvinyl alcohol and allowed to sediment for 30 min. Upper layer was collected, centrifuged (280 g, 10 min, RT) and residual erythrocytes were lysed. Isolated neutrophils were resuspended in HBSS buffer. Neutrophils (10^6^/reaction) were incubated with 40 µg of BK (24 h in 37ºC) while being stimulated with 5 µg/ml PMA (MedChemExpress) in the presence of elastase inhibitor, 1 mM PMSF (Merck). Respective controls with or without cells, peptide (BK and cBK) and PMA/PMSF were made for each set of samples. Cells were removed by centrifugation (400 g, 8 min, RT) and cold TCA was added to supernatant up to 15%. Samples were incubated on ice for 20 min, centrifuged (20 000 g, 10 min, 4ºC), and loaded onto HPLC column.

### HPLC

Reaction products were loaded onto the Aeris 3.6 µm PEPTIDE XB-C18 (150 × 4.6 mm) column (Phenomenex) and separated using NexeraX2 system in a rising gradient of 10% Phase A (0.1% TFA in water) to 40% Phase B (80% acetonitrile, 0.08% TFA) at the flow rate of 1.5 ml/min. Absorbance chromatograms (215 nm) were evaluated with the LabSolutions software.

### Cell culture

HaCaT (spontaneously immortalized human keratinocyte cell line, generously provided by dr Ewa Bielecka of the Department of Microbiology, Jagiellonian University, Kraków, Poland) cells were cultured in DMEM, 10% fetal bovine serum (FBS), 100 U/ml penicillin and 100 µg/ml streptomycin in 37 °C/5% CO2 and verified for absence of mycoplasma with the MycoAlert® Mycoplasma Detection Kit** (**Lonza). HBEC-5i (human brain endothelial cell line-5i, ATCC CRL-3245) were cultured on collagen-coated (Advanced BioMatrix) bottles in DMEM/F12 medium, 10% FBS, Endothelial Cell Growth Supplement (Cell Applications, Inc), 100 U/ml penicillin and 100 µg/ml streptomycin in 37 °C/5% CO2.

### Intracellular calcium flux

HaCaT cells were seeded onto 96-well plates and, upon reaching full confluence, were starved for 3 h (medium without FBS). Medium was removed and 80 µl of Calcium Dye 5 (Molecular Devices) diluted 10 times in HBSS with 20 mM HEPES pH 7.7, with or without B_2_ receptor inhibitor (3 µM HOE140) was added. After 1.5 h in 37 °C, fluorescence intensity (485/525 nm) was recorded continuously for 5 min using FlexStation 3 (Molecular Devices). At 17 s cells were stimulated with 0.001–500 nM BK or 0.5–1,500 nM cBK. FBS (3%) served as positive control. Results were calculated as area under the curves of RFU fold change from the signal recorded at baseline and adjusted to % of maximum response (FBS) curve. Non-linear regression analyses were performed to generate dose–response curves and calculate EC_50_ values.

### Western blot

Fully confluent HaCaT cells were starved overnight and subsequently incubated with either B_1_ or B_2_ receptor inhibitor (2 µM R715 or 2 µM HOE140) for 30 min. BK or cBK at 500 nM were then added for 15 min at 37 °C. Cells were washed and lysed in RIPA buffer (Merck) with cOmplete, Mini, EDTA-free Protease Inhibitor Cocktail (Roche) and PhosSTOP (Roche). Protein concentration was measured with the BCA Assay Kit (ThermoFisher). Fifty µg of protein was separated via SDS-PAGE and transferred onto PVDF. Membranes were blocked for 2 h in RT with 5% skim milk in the TBST buffer (50 mM Tris, 150 mM NaCl, 0.05% Tween 20), and then probed with primary antibodies (1:1500 in blocking buffer; T1325, Merck) against phosphotyrosine (pTyr) residues overnight in 4 °C. Membranes were incubated with goat anti-rabbit HRP-conjugated antibodies (1:5000; A0545, Merck) for 1 h in RT. Blots were washed, incubated with ECL Western Blotting Substrate, and signal was recorded on photographic film. Membranes were stripped (200 mM glycine, 0.1% SDS, 1% Tween-20, pH 2.2) for 10 min, washed, re-blocked and probed with mouse anti-β-actin antibodies (1:2000; A5441, Merck) for 1.5 h in RT. Membranes were incubated with goat anti-mouse HRP-conjugated antibodies (1:3000; A0168, Merck) for 1.5 h in RT, and developed.

### Wound healing

HaCaT cells were seeded onto culture inserts (Ibidi) in DMEM, 2.5% FBS. After 24 h, inserts were removed, cells were washed twice with DMEM and preincubated with 2 µM HOE140 for 30 min. Cells were stimulated with 500 nM BK or cBK, or 12.5 ng/ml EGF. Cell migration was documented at 0, 8, 16, 24, 48 and 72 h, and analysed with TScratch [46].

### Confocal microscopy

HaCaT cells were seeded onto μ-dishes with culture inserts (Ibidi). 24 h after wound healing experiment start, cells were washed thrice with PBS containing 1 mM CaCl_2_ (PBS +) and fixed with 4% paraformaldehyde (PFA) for 10 min at RT. Samples were then washed with PBS + , permeabilized with 0.1% Triton-X100/PBS + (BK receptors buffer) or with 0.1% saponin/PBS + (MMPs buffer) for 15 min in RT. Cells were blocked for 45 min in 1% BSA in PBS + (BK receptors) or 1% BSA in MMPs buffer. Cells were probed with the primary antibodies in blocking solution for 2 h at the room temperature; anti-B_1_ receptor rabbit antibody (1:200; ab75148, abcam), anti-B_2_ receptor mouse antibody (1:50; 610,452, BD Biosciences), anti-MMP9 (pro-form, active form) rabbit antibody (1:200; ab38898, abcam) or anti-MMP2 (pro-form) mouse antibody (1:50; MA5-13,590, ThermoFisher). Samples were incubated with the secondary antibodies for 1 h in RT: anti-rabbit AlexaFluor 647 (1:200; A-21244, ThermoFisher) or anti-mouse AlexaFluor 488 (1:200; A-11001, ThermoFisher), and then nuclei were counterstained with 1 mg/ml DAPI in PBS + for 10 min RT. The specificity of secondary antibodies was confirmed in the absence of primary antibody (data not shown). Images were acquired with 40x (N.A. 1.1) HC PL APO motCORR CS2 (BK receptors) or 20x (N.A. 0.75) HC PL APO CS2 (MMPs) objectives using the Leica TCS SP8 STED 3X. The Fiji [47] software and Dotted Line plugin (by Kevin Gali Baler) were used to draw the wound boundaries.

### RNA isolation and reverse transcription

HaCaT cells were starved for 2 h and pre-incubated with 2 µM HOE140 for 30 min. FBS-free medium was added with 500 nM native and cBK. After 4 h, cells were washed with PBS and lysed using TRIzol Reagent (ThermoFisher). RNA was isolated according to the manufacturer’s protocol, resuspended in 20 µl Rnase free water. A 1000 ng of RNA was used for cDNA synthesis using the High-Capacity cDNA Reverse Transcription kit (ThermoFisher).

### Real-time PCR

Five µl containing 8 ng cDNA were mixed with primers (Table 1) at the final concentration of 0.33 µM each, Power Up SYBR Green MasterMix (ThermoFisher) and water, to the final reaction volume of 15 µl.

**Table 1:** Primer sequences

Gene	Forward (5’- > 3’)	Reverse (5’- > 3’)
*EF2*	GACATCACCAAGGGTGTGCAG	TTCAGCACACTGGCATAGAGGC
*IL6*	CAGGAGCCCAGCTATGAACT	GAAGGCAGCAGGCAACAC
*IL8*	AGACAGCAGAGCACACAAGC	AGGAAGGCTGCCAAGAGAG
*VEGF-A*	CGGTGTCTGTCTGTGTGTC	AAGAGGAAAGAGGTAGCAAGAG

Real-time qPCR was performed as follows: 5 min at 95 °C; 40 cycles: 30 s at 95 °C, 30 s at 58 °C; 30 s at 72 °C. Reaction products were evaluated via melting curve. The relative fold change of expression was calculated via ΔΔC_T_.

### ELISA

Supernatants were collected at 24 h post-peptide stimulation (peptides: 500 nM, HOE140 2 µM). Concentration of IL6, IL8 and VEGF was assessed using DuoSet ELISA kits (RnD) following the manufacturer’s instructions. Results were normalized to cytokine concentration in unstimulated controls.

### Angiogenesis

HBEC-5i cells (1 mln/ml) were incubated with the CellTrace CFSE dye (5 µM; ThermoFisher) in DMEM/F12 for 45 min in 37 °C. Dye was then inactivated (5% FBS), cells washed and seeded on a collagen-coated bottle in fully supplemented media overnight. Cells were re-seeded (20,000 per well) onto reduced-growth factor GelTrex-coated (ThermoFisher) µ-plate (96 well; Ibidi) in the final volume of 70 µl DMEM/F12 phenol-free. Medium was supplemented with 0.5% FBS, with or without 1 µM BK or cBK. Images were acquired at 4h30 min with 4 × objective using Cytation 5 Cell Imaging Multimode Reader (BioTek). Nodes were counted manually by a non-blinded assessor using Fiji software.

### Animal model

Experiments were performed in accordance with the European legislation for the care and use of laboratory animals and in accordance with the permission obtained from the 1st Local Ethical Committee in Krakow, Poland (204/2022). Eight-week-old C57BL/6 female mice purchased from Janvier Labs (France) and were kept in specific-pathogen-free conditions, in 12 h night/12 h day cycles, in individually ventilated cages, with food and water ad libitum. Mice were anesthetized by intraperitoneal injection of 100 mg/kg ketamine/10 mg/kg xylazine and full-thickness wounds were induced on the back with a sterile punch biopsy needle (5-mm diameter). Each wound was treated with 10 µl of PBS or 100 nM peptide (BK/cBK) and after 24 h tissues from the wound periphery were collected.

### Immunohistochemistry

Dissected tissue was fixed in 4% PFA overnight, parafilm-embedded, sectioned and transferred onto glass slides. Samples were deparaffinized in xylene and rehydrated in ethanol (100–70%). Sections were incubated with picro-Sirius red solution for 1 h, then washed with acetic acid. Samples were dehydrated in ethanol (70–100%), cleared twice in xylene and mounted. Images were obtained using ScanScope™ system (Aperio) and viewed using the NDP.view 2 Imaging software (Hamamatsu Photonics K.K).

### Statistical analysis

Results are presented as means ± SEM of at least 3 independent experiments. Statistical significance was evaluated with t-test and one-Way ANOVA with Dunnett or Sidak post hoc. The differences were considered significant: * if *p* < 0.05, ** if *p* < 0.01 or *** if *p* < 0.001. The statistical analysis was performed using GraphPad Prism 9 software.

## Supplementary Information

Additional file 1: Fig. S1-S2. Fig. S1 – Original western blot image of the phosphorylated tyrosine residues shown in the Fig. 2E. Fig. S2 – Original western blot image of the β-actin shown in the Fig. 2E.

## Data Availability

All data needed to evaluate the conclusions in the paper is included in this published article and its Additional Files. Additional data related to this article will be made available from the corresponding author upon reasonable request.

## Abbreviations

ACE1: Angiotensin-converting enzyme 1
B_1_R: Bradykinin receptor 1
B_2_R: Bradykinin receptor 2
BK: Bradykinin
CPM: Carboxypeptydase M
HBEC-5i: Human brain endothelial cell line-5i
HMWK: High-molecular-weight kininogen
KKS: Kinin-kallikrein system
MMP: Matrix metalloproteinase
NEP: Neprilysin
