# Supplementary material for: Bradykinin’s carbamylation as a mechanistic link to impaired wound healing in patients with kidney dysfunction
Source: BMC Biol. 2025 Mar 12;23:76. doi: 10.1186/s12915-025-02187-x (PMC11905624; doi:10.1186/s12915-025-02187-x)
Supplement: Supplementary file 1 — Additional file 1: Fig. S1-S2. Fig. S1 – Original western blot image of the phosphorylated tyrosine residues shown in the Fig. 2E. Fig. S2 – Original western blot image of the β-actin shown in the Fig. 2E. [file 12915_2025_2187_MOESM1_ESM.pdf]

Fig S1

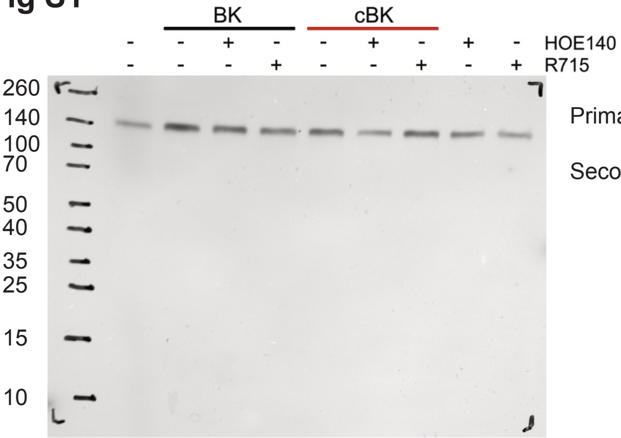

Primary antibody: anti-pTyr 1:1500

Secondary antibody: anti-rabbit HRP 1:5000

Fig S2

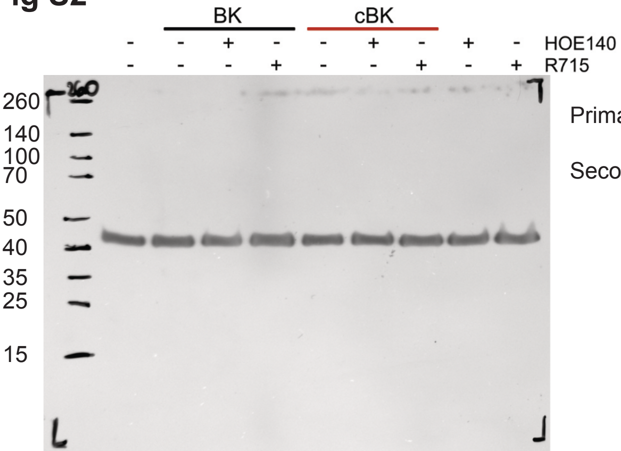

Primary antibody: anti-β-actin 1:2000

Secondary antibody: anti-mouse HRP 1:3000
